# cMeta-INR: cohort-informed meta-learning-based implicit neural representation for deformable registration-driven real-time volumetric MRI estimation

**DOI:** 10.1088/1361-6560/ae29e2

**Published:** 2025-12-22

**Authors:** Xiaoxue Qian, Hua-Chieh Shao, Jing Cai, You Zhang

**Affiliations:** 1 The Medical Artificial Intelligence and Automation (MAIA) Laboratory and Department of Radiation Oncology, University of Texas Southwestern Medical Center, Dallas, TX 75390, United States of America; 2 Department of Health Technology & Informatics, The Hong Kong Polytechnic University, Hong Kong Special Administrative Region of China, People’s Republic of China

**Keywords:** deformable image registration, implicit neural representation (INR), meta learning, population prior, test-time adaptation

## Abstract

*Objective.* Rapid and accurate reconstruction of high-quality three-dimensional magnetic resonance (MR) images from undersampled *k*-space data with variable sampling patterns remains a challenge due to limited available information and the need to preserve rich anatomical details. Deformable image registration provides a promising solution by warping a fully-sampled reference image to align with undersampled data acquired on-board (from an image-guided treatment delivery platform like MR-LINACs). In this study, we proposed a novel registration framework—cohort-informed meta-learning (cMeta)—to enhance the accuracy and efficiency of implicit neural representations (INR) for limited *k*-space data-driven patient-specific deformable image registration. *Approach.* cMeta-INR incorporated token-aware modulation and population-level deformation priors to guide an INR template-based meta-learning process. By encoding contextual information and leveraging cohort-informed priors, the resulting meta-learning framework enabled the INR to rapidly adapt to new registration cases with undersampled *k*-space data. Specifically, for the meta learning, a modulation module with token-awareness was introduced to modulate the INR template, and a pre-trained population-based registration network (KS-RegNet) was employed to generate coarse, reference deformation vector fields and latent embeddings for computing the deformation discrepancy loss and embedding similarity loss. During test-time adaptation, the INR, initialized from the meta-learned template, was efficiently fine-tuned to new *k*-space data with minimal iterations. *Main results.* Experiments were conducted on 14 abdominal and 11 cardiac 4D magnetic resonance imagings (MRIs) with 5–13 spokes. cMeta-INR outperformed state-of-the-art methods, achieving the best average (± s.d.) Dice similarity coefficients (0.778 ± 0.056 for abdominal and 0.894 ± 0.076 for cardiac data), and center-of-mass errors (3.04 ± 1.48 mm and 1.32 ± 1.02 mm, respectively), while enabling rapid test-time adaptation of only ∼35 s on an NVIDIA H100 GPU. *Significance.* The proposed cohort-informed meta-learning framework effectively enhanced the adaptation capabilities of INRs to individual patients under highly undersampled *k*-space scenarios, demonstrating strong potential for fast and accurate patient-specific deformable registration.

## 1. Introduction

With high-resolution anatomical and functional imaging capabilities, superior soft-tissue contrast, and zero ionizing radiation, three-dimensional (3D) magnetic resonance imaging (MRI) has become indispensable in medical diagnosis and treatment (Wagner and Conti 1991, Schmidt and Payne 2015). However, the inherently slow acquisition of fully-sampled *k*-space data limits its applicability in time-sensitive clinical scenarios—for example, motion-resolved imaging and real-time image-guided applications (Corradini *et al* 2019, Talanki *et al* 2022, Cruz *et al* 2023). To accelerate acquisition, MR imaging often resorts to undersampled *k*-space data. This undersampling violates the Nyquist–Shannon sampling criterion, frequently resulting in aliasing artifacts in the reconstructed images (Ravishankar and Bresler 2010, Knoll *et al* 2020, Wang *et al* 2021). Although various reconstruction methods, such as compressed sensing (Lustig *et al* 2007, Geethanath *et al* 2013), dictionary learning (Ravishankar and Bresler 2010, Zhan *et al* 2015), and deep learning-based techniques (Liu *et al* 2018, Wang *et al* 2020, 2021), have been proposed to mitigate artifacts like blurriness and ghosting, rapidly and accurately restoring anatomically faithful images from sparse *k*-space data remains a challenge.

Instead of relying on reconstruction algorithms to recover images from undersampled *k*-space data, deformable image registration leverages deformation vector fields (DVFs) to warp a fully-sampled prior image, enabling the estimation of high-quality images that are consistent with the undersampled measurements (Qin *et al* 2019, Han *et al* 2022). This approach is particularly advantageous in workflows such as image-guided radiotherapy (Rivaz *et al* 2014, Rigaud *et al* 2019), where fully-sampled pre-treatment reference images can be leveraged to enhance image quality and anatomical consistency without requiring full acquisitions at each time. Several studies have explored this paradigm to improve MR imaging quality. Stemkens *et al* proposed a model-based motion estimation approach (2016) that employed a principal component analysis (PCA)-based motion model to estimate 3D DVFs from 2D MRIs. These DVFs were subsequently used to deform a prior 3D image and estimate motion-resolved 3D MR images. Shao *et al* proposed KS-RegNet (2022), a deep learning model in which the deformation-related data fidelity loss was evaluated directly in the *k*-space domain using an unsupervised training scheme. The model achieved competitive registration performance without relying on ‘ground-truth’ DVF labels. Ghoul *et al* proposed LAPANet (2024), a self-supervised learning framework designed to estimate deformable motion from undersampled *k*-space data. By combining local all-pass motion modeling with Transformer-based attention mechanisms, LAPANet yielded enhanced accuracy compared to both conventional and deep learning-based registration methods. These studies demonstrate the potential of deformable image registration in estimating high-quality images from undersampled *k*-space data. Nevertheless, in capturing fine-scale deformations, existing feed-forward learning-based registration frameworks often lack flexibility and generalizability when applied to diverse subjects with potential distribution shifts, leading to compromised accuracy (Hansen and Heinrich 2021).

Coordinate-based implicit neural representations (INRs) have recently been employed for deformable image registration (Wolterink *et al* 2022, Byra *et al* 2023, van Harten *et al* 2024). In this paradigm, a lightweight neural network is trained to approximate the DVFs by mapping input spatial coordinates to their corresponding displacement vectors, based on a loss function defined on image similarity metrics. The case-specific optimization nature of INR-based learning matches that of conventional iterative registration algorithms, rendering it robust to inter-case variability (Sitzmann *et al* 2020, Xu *et al* 2023). A range of INRs have been proposed, such as cycle-consistent INR (Van Harten *et al* 2023), spline-enhanced INR (Sideri-Lampretsa *et al* 2024), INR with neural velocity field (Han *et al* 2023), and geometry-informed INR (van Harten *et al* 2024), to enforce cycle-consistency loss, promote smooth deformations, and integrate structural priors in deformable image registration. Compared to traditional optimization-based methods, INRs achieve fast convergence through stochastic gradient descent. It also offers a continuous and resolution-independent formulation of deformation, enabling accurate modeling of complex anatomical motion. In contrast to deep learning-based models, INRs are more lightweight and not dependent on large training datasets (Grattarola and Vandergheynst 2022, Khan and Fang 2022, Gielisse and van Gemert 2025), as they can be trained on each case via ‘one-shot’ learning. Despite these advantages, optimizing INRs from scratch for each new case during inference inevitably faces a trade-off between computational efficiency and representation capacity, making them difficult to deploy in time-sensitive clinical workflows (Molaei *et al* 2023). Moreover, the limited representation capacity of shallow MLPs may hinder the capture of fine anatomical details and constrain the registration accuracy.

To accelerate inference and boost the registration accuracy of INRs, recent studies have explored meta-learning approaches that learn generalizable initialization parameters across multiple patients for the INRs (Lee *et al* 2021, Chen and Wang 2022, van Harten *et al* 2024, Yang *et al* 2025). By accumulating and transferring knowledge from multiple patient registration tasks, meta-learning enabled the INRs to rapidly adapt to new case-specific data with only a few optimization steps. Van Harten *et al* proposed REINDIR (2024), a meta-learning framework that combined an image encoder with a population-based INR template, where the image embedding output of the image encoder will modulate the INR template to specialize it for a test image pair. This specialization provided a better initialization of INRs for the subsequent optimization process. Another strategy for enhancing the performance of INRs involves incorporating cohort-level anatomical priors from a pre-trained population-based registration model into the model optimization process (Han *et al* 2023). For example, Qian *et al* proposed a hybrid population-based and patient-specific framework for deformation-driven cone-beam CT estimation (2025), where a pre-trained, population-based deep learning network was used to generate reference DVFs to guide the INR optimization at test time. By leveraging population-informed priors, this approach alleviates the inherent trade-off between optimization efficiency and registration accuracy in patient-specific 2D–3D deformable image registration.

To address the limitation of slow acquisition in time-sensitive clinical scenarios and enable accurate volumetric MRI for real-time image-guided applications such as motion compensation and treatment guidance in adaptive radiotherapy, we propose cMeta-INR, a cohort-informed meta-learning framework designed to boost INRs. This framework incorporates cohort-level priors as regularizations into the meta-learning process to optimize an INR template, thereby enhancing INR registration accuracy and facilitating rapid adaptation to new patients, enabling real-time volumetric MRI during treatments to guide motion-aware adaptive radiotherapy. In our approach, a token-aware modulator was designed to capture contextual information from the to-be-registered image pair, which was then used to generate structured modulation coefficients for modulating the INR template. Meanwhile, population-level DVFs and embeddings, derived from a pre-trained, deeper registration network (KS-RegNet) (Shao *et al* 2022), were employed to guide the meta-learning of the INR template. In general, our meta-learning mechanism was cohort-based, utilizing a population dataset for training, while test-time inference was performed via case-specific adaptation/optimization. During test-time adaptation, the INR was initialized from the learned INR template and rapidly fine-tuned on new, highly undersampled *k*-space data using four loss functions: deformation discrepancy between KS-RegNet- and INR-predicted DVFs, *k*-space fidelity between the deformed source volume and the target image, embedding similarity between the deformed source volume and the target image, and DVF smoothness regularization. The primary contributions of this study are summarized as follows:

1) A token-aware modulator was developed to generate structured modulation coefficients, enabling fine-grained modulation of the INR template.
2) The proposed cohort-informed meta-learning framework exploited population-level deformation priors and embeddings to guide the meta-learning of an INR template, facilitating rapid and accurate patient-specific, registration-driven 3D MRI estimation from highly undersampled *k*-space data during the test time.
3) By integrating deformation discrepancy, *k*-space fidelity, embedding similarity, and smoothness regularization into both meta-training and test-time adaptation, cMeta-INR achieved superior registration accuracy with minimal test-time optimization for specific test subjects.

## 2. Materials and methods

**Figure 1.**
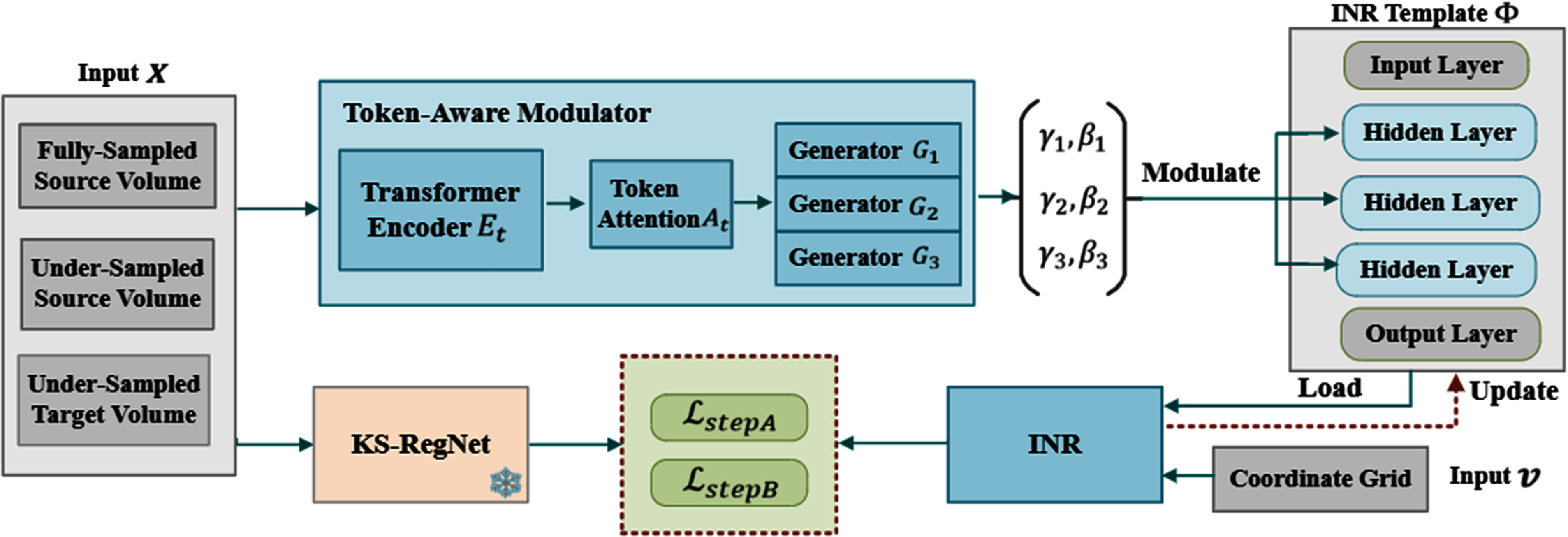
Overview of the cohort-informed meta-learning framework for learning the INR template ${{\Phi }}$. The token-aware modulator generates the modulation coefficients ($\gamma ,{\text{ }}\beta $) to adjust the hidden layers of the INR template. The pre-trained KS-RegNet (frozen) predicts the DVFs used to optimize the INR in step A, with the loss ${\mathcal{L}_{{\text{stepA}}}}$. During the meta-learning process, new training data are continuously fed into the corresponding inputs of the model, which inherits/loads the existing INR template and updates it through a two-step optimization scheme using ${\mathcal{L}_{{\text{stepA}}}}$ and ${\mathcal{L}_{{\text{stepB}}}}$.

To accelerate the optimization of INRs for patient-specific deformable image registration, we proposed cMeta-INR: a cohort-informed meta-learning framework designed to enhance the representation capacity and enable faster convergence of INRs for new cases. The overall algorithm comprised two key stages: cohort-informed meta-learning (figure 1), which optimized a generalized INR template, and patient-specific adaptation (figure 3), where the INR inherited the learned template and was further fine-tuned to fit new patient data.

### 2.1. Cohort-informed meta-learning

Since training an INR from scratch was computationally intensive for test-time optimization of 3D DVFs, meta-learning mechanisms such as Reptile (Nichol *et al* 2018) and REINDIR (van Harten *et al* 2024) were proposed. In this paradigm, the meta-learning model enables INRs to acquire a generalizable, initial template which is then iteratively updated by taking a few small steps toward the task-specific optimum, allowing rapid adaptation to new patient data. However, due to the lack of prior information and the shallow network structure, existing meta-learning-based INR approaches still struggle with adaptability and generalization to unseen cases, particularly when meta-training data is limited (Yao *et al* 2021). To address these challenges, we proposed a cohort-informed meta-learning, as shown in figure 1, it was guided by population-level deformation priors and embeddings obtained from a pre-trained, deeper registration network. Moreover, we designed a token-aware modulator that enabled fine-grained knowledge extraction by generating structured modulation coefficients to guide the INR optimization.

**Figure 2.**
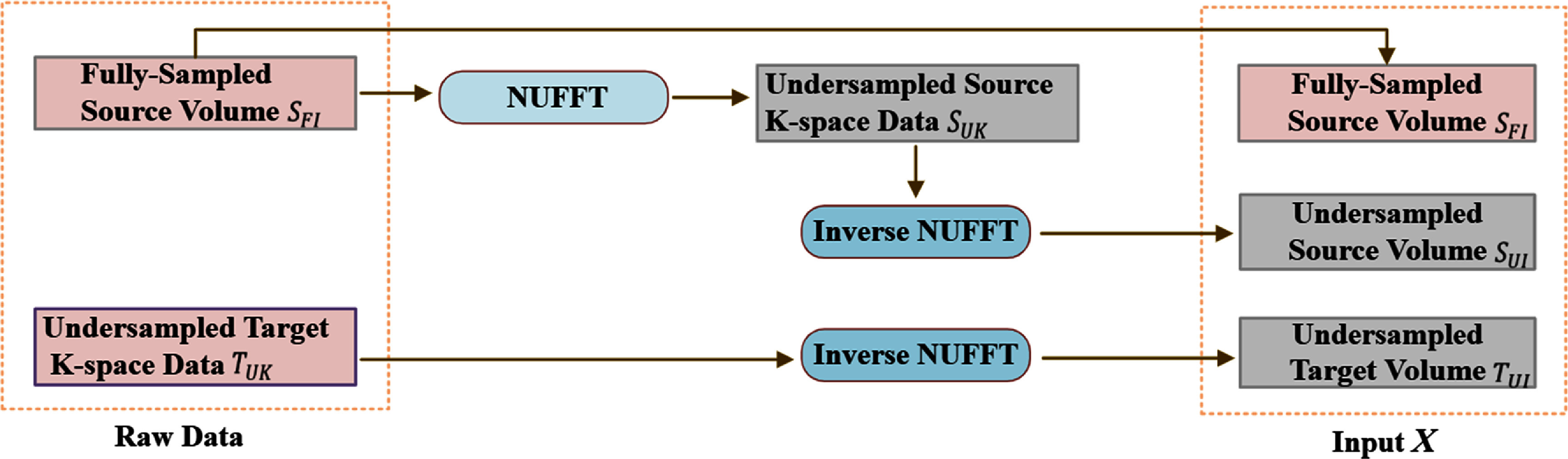
Overview of the input preprocessing pipeline. It converts the two original/raw data $({S_{{\text{FI}}}},{ }{T_{{\text{UK}}}}{\text{ )}}$ into three input volumes (${S_{{\text{FI}}}},{S_{{\text{UI}}}},{ }{T_{{\text{UI}}}}{ }$). Specifically, the fully sampled source volume ${S_{{\text{FI}}}}$ is first transformed into the undersampled source *k*-space data ${\text{ }}{S_{{\text{UK}}}}$, followed by an inverse NUFFT to obtain the undersampled source volume ${S_{{\text{UI}}}}$. The undersampled target *k*-space data ${T_{{\text{UK}}}}$ is reconstructed into the undersampled target volume ${T_{{\text{UI}}}}$ via inverse NUFFT.

Following a similar training strategy to KS-RegNet (Shao *et al* 2022), the raw input data for our meta-learning framework comprised a fully-sampled prior source image ${S_{{\text{FI}}}} \in {C^{2 \times H \times W \times D}}$ (2 channels for the real and imaginary parts) and on-board undersampled *k*-space data ${T_{{\text{UK}}}}$ (target). To preprocess and align these data for model input, the target *k*-space data were reconstructed into spatial-domain images using the inverse non-uniform fast Fourier transform (NUFFT) (Muckley *et al* 2020), resulting in a complex-valued, undersampled target image ${T_{{\text{UI}}}} \in {C^{{{2 \times }}H{ \times }W{ \times }D}}$. In addition, following the same logic of the previous KS-RegNet study (Shao *et al* 2022), the fully-sampled source image ${S_{{\text{FI}}}}$ was intentionally projected into undersampled *k*-space data ${S_{{\text{UK}}}}$ by applying NUFFT with the same radial readout trajectory as the target acquisition (${T_{{\text{UK}}}}$). To perceive undersampling artifacts matching those of ${T_{{\text{UI}}}}$, the resulting *k*-space data ${S_{{\text{UK}}}}$ was reconstructed via inverse NUFFT to obtain an undersampled source image ${S_{{\text{UI}}}}$ $ \in {C^{2 \times H \times W \times D}}$, which served as an additional input. The final input to the model consisted of the concatenation of the real and imaginary parts of the three complex-valued images *X* $ = \left( {{S_{{\text{FI}}}},{ }{S_{{\text{UI}}}},{ }{T_{{\text{UI}}}}} \right)$, resulting in a six-channel input ${\text{X}} \in {{\text{R}}^{6 \times {\text{H}} \times {\text{W}} \times {\text{D}}}}$. The overall input preprocessing workflow for generating the model input is illustrated in figure 2.

As shown in figure 1, the token-aware modulator, integrated within our cMeta-INR framework, consists of three components: a transformer encoder ${E_{{t}}}$ that extracts contextual tokens from the inputs *X*; a token attention layer ${A_{{t}}}$ that functionally decouples the extracted tokens to focus on modulation-relevant information; and three modulation coefficient generators $\left( {{G_1},{ }{G_{2,{ }}},{G_3}} \right)$ that translate the attentional tokens into structured, layer-wise modulation parameters $\left\{ {\left( {{\gamma _1},{\beta _1}} \right),{ }\left( {{\gamma _2},{\beta _2}} \right),{ }\left( {{\gamma _3},{\beta _3}} \right)} \right\}$. The INR template is defined as ${\boldsymbol{\Phi }} = \left\{ {\left( {{w_i},{b_i}} \right),\left( {{w_{l\,}},{b_l}} \right),{ }\left( {{w_o},{b_o}} \right)} \right\}$, where *i, l* $ \in \left\{ {1,{\text{ }}2,{\text{ }}3} \right\}$, and *o* indicate the input, hidden (three), and output layers, respectively. $w$ represents the neuron weightings and $b$ denotes the bias. In our method, only the hidden layers were modulated. The modulation of the INR template is operated by:

\begin{equation*}{F^{\left( {\text{l}} \right)}} = \sigma \left({\gamma_{\text{l}}} \cdot \left({w_{\text{l}}} \cdot {F^{\left( {{\text{l}} - 1} \right)}}\right) + {\beta _{\text{l}}} \cdot {b_{\text{l}}}\right),\end{equation*}

where ${ }{F^{\left( {\text{l}} \right)}}$ represents the output of the $l$th hidden layer, with ${F^{\left( 0 \right)}}$ corresponding to the output of the input layer, and $\sigma $ denotes the SIREN activation function (Sitzmann *et al* 2020).

### 2.2. Case-specific adaptation

**Figure 3.**
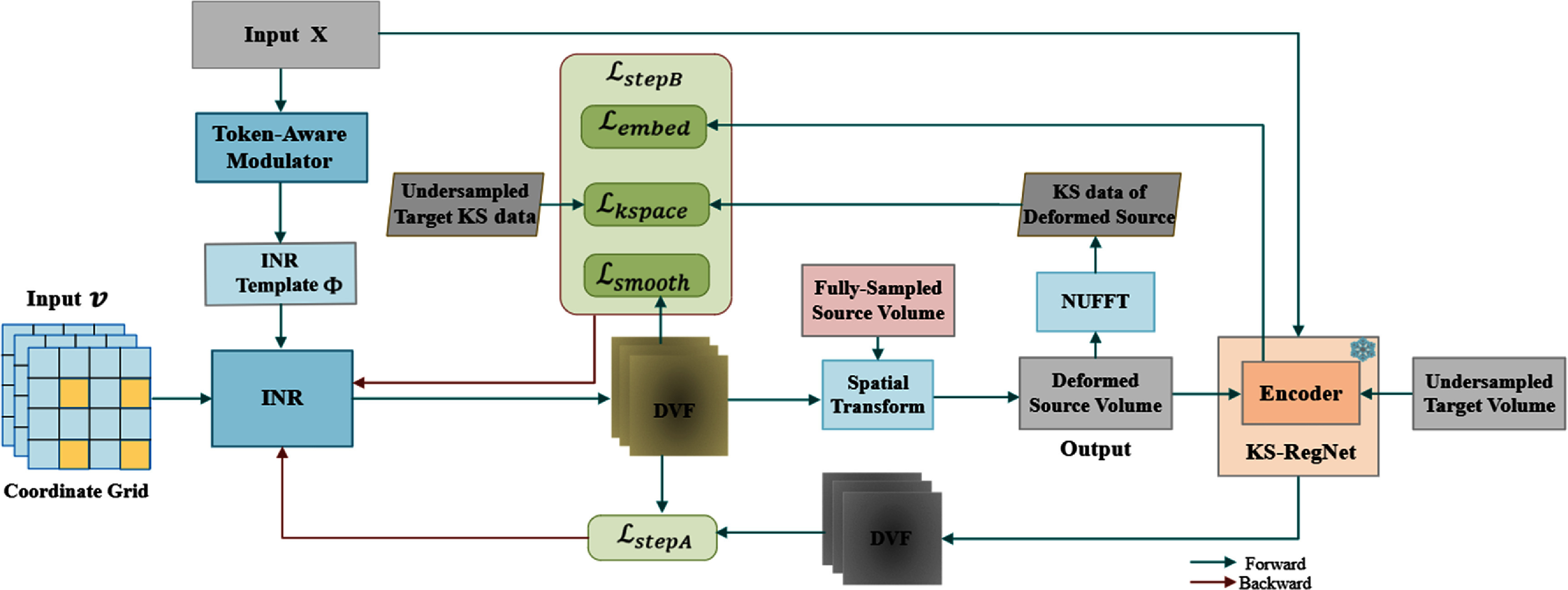
Case-specific adaptation, where the INR initialized via the learned INR template ${{\Phi }}$ was further optimized for a new patient registration case using four loss functions: deformation discrepancy loss ${\mathcal{L}_{{\text{stepA}}}}$, *k*-space fidelity loss ${\mathcal{L}_{k{\text{space}}}}$, embedding similarity loss ${\mathcal{L}_{{\text{embed}}}}$, and deformation smoothness loss ${\mathcal{L}_{{\text{smooth}}}}$. The pre-trained KS-RegNet (frozen) predicts the DVFs used to calculate ${\mathcal{L}_{{\text{stepA}}}}$, the encoder of KS-RegNet is employed to extract feature embeddings for calculating the ${\mathcal{L}_{{\text{embed}}}}$, and ${\mathcal{L}_{k{\text{space}}}}$ is computed between the undersampled target *k*-space data and the corresponding *k*-space data of the deformed source image.

Figure 3 illustrates the case-specific adaptation stage, in which the cMeta-INR model is adapted to each patient-specific registration case by fine-tuning the parameters inherited from the meta-learned template ${\boldsymbol{\Phi }}$, using two steps of loss functions described in section 2.3. To reduce computational complexity, the spatial coordinates $v = \left( {x,y,z} \right) \in {R^3}$, representing voxel locations in Cartesian space, were downsampled and input to the INR, which output the corresponding voxel-wise DVF ${\widehat {\mathbf{d}}_{{\text{inr}}}}\left( {\mathbf{v}} \right)$. The predicted DVF was subsequently upsampled to the original resolution and applied to the fully-sampled prior (source image ${S_{{\text{FI}}}}$) via a spatial transformation layer (Balakrishnan *et al* 2019) to obtain the deformed source image ${\hat S_{{\text{DI}}}}$. This deformed image was then projected into the *k*-space domain using NUFFT, yielding the *k*-space data ${{{\hat S}}_{{\text{DK}}}}$ of the deformed source image that can be compared with the target *k*-space data ${T_{{\text{UK}}}}{ }$ to guide INR optimization. Importantly, this test-time adaptation procedure was fully unsupervised and did not require ‘ground-truth’ DVFs, making it suitable for deployment in clinical settings with only undersampled *k*-space measurements.

### 2.3. Loss function design and training procedures

The cMeta-INR framework comprised two stages: prior meta-learning for optimizing the INR template, and test-time adaptation for case-specific DVF inference. Both stages follow a two-step optimization scheme (step A and step B). For step A, a pre-trained, UNet-based KS-RegNet (Shao *et al* 2022) was used to predict the DVF ${\widehat {\mathbf{d}}_{{\text{reg}}}}\left( {\mathbf{v}} \right){\text{ }}$ as the population-based reference for optimizing the INR via the mean absolute error loss function, denoted as cohort-informed deformation discrepancy loss:

\begin{equation*}{\mathcal{L}_{{\text{stepA}}}} = \sum\limits_{j = x,y,z} {\frac{1}{N}} \sum\limits_{i = 1}^N {\left| {{{\hat d}_{{\text{inr}},j}}\left( {{\boldsymbol{v}_i}} \right) - {{\hat d}_{{\text{reg}},j}}\left( {{\boldsymbol{v}_i}} \right)} \right|} ,\end{equation*}

where $N$ denotes the total voxel number of the DVF and $j$ denotes the Cartesian direction.

For step B, three loss functions were used to update the parameters of INR. We used the *k*-space fidelity loss to measure the similarity between the NUFFT-projected *k*-space data ${{{\hat S}}_{{\text{DK}}}}$ of the deformed source image (${{{\hat S}}_{{\text{DI}}}}$) and the undersampled target *k*-space data ${T_{{\text{UK}}}}$, and calculated the mean squared error between the two complex-valued *k*-space data as the *k*-space fidelity loss:

\begin{equation*}{\mathcal{L}_{{\text{kspace}}}} = \frac{1}{{{N_{\text{k}}}}}\sum\limits_{i = 1}^{{N_{\text{k}}}} {{{\left| {{{\hat S}_{{\text{DK}}}}\left( {{{\text{k}}_i}} \right) - {{{T}}_{{\text{UK}}}}\left( {{{\text{k}}_i}} \right)} \right|}^2}} ,\end{equation*}

where ${N_k}$ is the number of sampled points in *k*-space, and ${k_i}$ represents the *i*th sampling frequency.

The *k*-space data typically concentrate in the low-frequency region due to MRI sampling patterns (Lustig *et al* 2008, Raja and Sinha 2014). To better capture high-frequency anatomical details, we introduced an embedding-space loss, which measured the semantic similarity between the latent space vectors of the deformed source image ${{{\hat S}}_{{\text{DI}}}}$ and the target image ${T_{{\text{UI}}}}$, termed embedding similarity loss. These embeddings were extracted using the encoder of the pre-trained KS-RegNet. The loss was formulated using the cosine similarity (Zhou *et al* 2024):

\begin{equation*}{\mathcal{L}_{{\text{embed}}}} = \frac{1}{{{N_e}}}\sum\limits_{i = 1}^{{N_e}} {\frac{{\boldsymbol{E}\left( {{{\hat S}_{{\text{DI}}}}} \right),\boldsymbol{E}\left( {{T_{{\text{UI}}}}} \right)}} {\left\|\boldsymbol{E}\left( {{{\hat S}_{{\text{DI}}}}} \right)\right\|.\|\boldsymbol{E}\left( {{T_{{\text{UI}}}}} \right)\|}} ,\end{equation*}

where ${\mathbf{E}}\left( {{{{{\hat S}}}_{{\text{DI}}}}} \right)$ and ${\mathbf{E}}\left( {{{{T}}_{{\text{UI}}}}} \right){\text{ }}$ denote the latent embeddings of the deformed source and the target volumes, respectively, ${\text{ }}{\mathbf{E}}$ is the encoder, and ${N_{\text{e}}}$ is the total number of embedding vectors. This loss encouraged semantic alignment in the feature space, helping preserve fine-grained structural information during registration.

A third loss function was introduced to further regularize the smoothness of the DVFs. It calculated the mean deformation energy of the DVF via:

\begin{equation*}{\mathcal{L}_{{\text{smooth}}}} = \sum\limits_{j = x,y,z} {\frac{1}{{\text{N}}}} \sum\limits_{{\text{i}} = 1}^{\text{N}} {\left( {{{\left( {\frac{{\partial {{\hat d}_j}\left( {{{\mathbf{v}}_{\text{i}}}} \right)}}{{\partial {\text{x}}}}} \right)}^2} + {{\left( {\frac{{\partial {{\hat d}_j}\left( {{{\mathbf{v}}_{\text{i}}}} \right)}}{{\partial {\text{y}}}}} \right)}^2} + {{\left( {\frac{{\partial {{\hat d}_j}\left( {{{\mathbf{v}}_{\text{i}}}} \right)}}{{\partial z}}} \right)}^2}} \right)} ,\end{equation*}

where ${{{\hat d}}_{\text{j}}}$ denotes a Cartesian component $j \in \left( {x,y,z} \right)$ of $\widehat {\mathbf{d}}\left( {\mathbf{v}} \right)$.

The total loss function in step B was a weighted sum of the three losses:

\begin{equation*}{\mathcal{L}_{{\text{stepB}}}} = {\lambda _1}{\mathcal{L}_{{\text{kspace}}}} + {\lambda _2}{\mathcal{L}_{{\text{embed}}}} + {\lambda _3}{\mathcal{L}_{{\text{smooth}}}}\end{equation*}

where ${\mathbf{\lambda }}$ denote the weighting hyper-parameters. In this work, ${\lambda _1}$ = 1.0, ${\text{ }}{\lambda _2}$ = 0.5, and ${\text{ }}{\lambda _3}$ = 0.5 were empirically determined by trial-and-error experiments.

In the meta-learning process, the token-aware modulator, which generated modulation coefficients to condition the INR, was updated. Specifically, after several epochs of step A followed by step B to optimize the INR on a given case via stochastic gradient descent, the parameters of the modulation module were updated through backpropagation. Meanwhile, the meta-parameters of the INR template were updated by averaging the differences between the post-training and initial parameters across multiple subjects, similar to Reptile (Nichol *et al* 2018). During test-time adaptation, only a few epochs of steps A and B were used to fine-tune the INR for a new registration case. The complete training and test-time optimization strategy is summarized in algorithm 1.

**Table:** 

**Algorithm 1.** cMeta-INR training and test-time adaptation.
**Input and Notation:**
${\tilde X_{{\text{train}}}}$ denotes the training dataset with ${{\text{N}}_{\text{x}}}$ cases. $\mathcal{M}$ is the token-aware modulator with parameters ${\boldsymbol{\theta }}$. ${\boldsymbol{\gamma }}$ and ${\boldsymbol{\beta }}$ are the generated modulation parameters, ${\mathbf{v}}$ is the input to the INR, ${\boldsymbol{\Phi }}$ is the INR template, and ${\boldsymbol{\Delta \varphi }}$ denotes its parameter update. ${{\text{N}}_{{\text{outer}}}} = 500,$ ${{\text{N}}_{{\text{inner}}}} = 10$, and ${{\text{N}}_{{\text{adapt}}}} = 50.$
**cMeta-INR training:**
**for** iteration = 1, 2, …, ${{\text{N}}_{{\text{outer}}}}$ do
${\boldsymbol{\Delta \varphi }} = 0$
**for** subject X in ${\widetilde {\mathbf{X}}_{{\text{train}}}}$ do
$\left( {{\mathbf{\gamma }},{\mathbf{\beta }}} \right)$ ← Frozen $\mathcal{M}$ (X; ${\boldsymbol{\theta }}$)
${\text{INR}}\left( {{\mathbf{v}};{\boldsymbol{\Phi }},{\boldsymbol{\gamma }},{\boldsymbol{\beta }}} \right)$ ←Load parameters ${\boldsymbol{\Phi }},$ ${\boldsymbol{\gamma }},{\boldsymbol{\beta }}$
**for** epoch =1, 2, …, ${{\text{N}}_{{\text{inner}}}}$ do
${{\boldsymbol{\Phi }}^{^{^{\prime}}}}{\text{ }}$ ← Update ${\text{INR}}\left( {{\mathbf{v}};{\boldsymbol{\Phi }},{\boldsymbol{\gamma }},{\boldsymbol{\beta }}} \right)$ with equation (2)
**end**
**for** epoch =1, 2, …, ${{\text{N}}_{{\text{inner}}}}$ do
${\text{ }}{{\boldsymbol{\Phi }}^{^{\prime\prime}}}$ ← Update ${\text{INR}}\left( {{\mathbf{v}};{\text{ }}{{\boldsymbol{\Phi }}^{\prime}},{\boldsymbol{\gamma }},{\boldsymbol{\beta }}} \right)$ with equation (6)
**end**
${{\Delta }}{\boldsymbol{\varphi }}$ ←${{\Delta }}{\boldsymbol{\varphi }} + ({{\boldsymbol{\Phi }}^{{^{^{\prime\prime}}}}} - {\boldsymbol{\Phi }})/{{\text{N}}_{\text{x}}}$
Frozen ${\text{INR}}\left( {{\mathbf{v}};{{\boldsymbol{\Phi }}^{^{\prime\prime}}},{\text{ }}{\boldsymbol{\gamma }},{\text{ }}{\boldsymbol{\beta }}} \right)$
Update $\mathcal{M}$ (X; ${\boldsymbol{\theta }}$) with equation (6)
**end**
${\boldsymbol{\Phi }}$ ←${\text{ }}{\boldsymbol{\Phi }} + {{\Delta }}{\boldsymbol{\varphi }}$
**end**
**Test-time adaptation:**
$\left( {{\boldsymbol{\gamma }},{\boldsymbol{\beta }}} \right)$ ← Frozen ${\text{ }}\mathcal{M}$ (**X_test_** $;{\mathbf{\theta }}$)
${\text{INR}}\left( {{\mathbf{v}};{\boldsymbol{\Phi }},{\boldsymbol{\gamma }},{\boldsymbol{\beta }}} \right)$ ← Load parameters ${\boldsymbol{\Phi }},{\boldsymbol{\gamma }},{\boldsymbol{\beta }}$
**for** epoch = 1, 2, …, ${{\text{N}}_{{\text{adapt}}}}$ do
${{\boldsymbol{\Phi }}^{{^{\prime}}}}{\text{ }}$ ← Update ${\text{ INR}}\left( {{\mathbf{v}};{\boldsymbol{\Phi }},{\boldsymbol{\gamma }},{\boldsymbol{\beta }}} \right)$ with equation (2)
**end**
**for** epoch =1, 2, …, ${{\text{N}}_{{\text{adapt}}}}$ do
${{\boldsymbol{\Phi }}^{{{^{\prime\prime}}}}}{\text{ }}$ ← Update ${\text{ INR}}\left( {{\mathbf{v}};{{\boldsymbol{\Phi }}^{{^{\prime}}}},{\boldsymbol{\gamma }},{\boldsymbol{\beta }}} \right)$ with equation (6)
**end**

### 2.4. Datasets

Two MRI datasets (abdominal and cardiac) were employed to evaluate the capability of cMeta-INR in solving patient-specific DVFs based on limited *k*-space sampling. The following sections describe the dataset specifications and initial preprocessing steps in detail.

#### 2.4.1. Abdominal dataset

The abdominal 4D-MRI dataset consisted of 14 subjects with liver tumors, including 5 in-house cases from the University of Texas Southwestern Medical Center and 9 cases shared by the Hong Kong Polytechnic University, which were acquired under an umbrella institutional review board protocol. The images were magnitude-only, without access to raw *k*-space data. The 5 in-house data are T2-weighted with a voxel size of 0.88 × 0.88 × 3.0 mm^3^. The remaining 9 data were acquired in planar mode and retrospectively sorted into 4D-MRI volumes, each with a slice thickness of 5 mm and 36–40 slices per respiratory bin, depending on the subject. These data are T1-weighted with an in-plane resolution of 256 × 256 and voxel sizes ranging from 1.41 × 1.41 mm^2^ to 1.88 × 1.88 mm^2^ across subjects. Except for one case (6 respiratory bins), each subject had 10 respiratory-resolved bins. The main acquisition parameters are summarized in table 1.

**Table 1.** MR imaging parameters of the subjects in the abdominal dataset.

Subject	Field-of-view (mm^3^)	Volume Size	Resolution (mm^3^)	No. of slices	No. of respiratory bins
1	480 × 480 × 180	256 × 256 × 36	1.88 × 1.88 × 5.0	36	6
2	480 × 480 × 200	256 × 256 × 40	1.88 × 1.88 × 5.0	40	10
3	480 × 480 × 200	256 × 256 × 40	1.88 × 1.88 × 5.0	40	10
4	480 × 480 × 215	256 × 256 × 43	1.88 × 1.88 × 5.0	43	10
5	360 × 360 × 200	256 × 256 × 40	1.41 × 1.41 × 5.0	40	10
6	480 × 480 × 300	256 × 256 × 60	1.88 × 1.88 × 5.0	60	10
7	420 × 420 × 240	256 × 256 × 48	1.64 × 1.64 × 5.0	48	10
8	360 × 360 × 210	256 × 256 × 42	1.41 × 1.41 × 5.0	42	10
9	480 × 480 × 200	256 × 256 × 40	1.88 × 1.88 × 5.0	40	10
10	450 × 450 × 105	512 × 512 × 53	0.88 × 0.88 × 3.0	53	10
11	450 × 450 × 105	512 × 512× 53	0.88 × 0.88 × 3.0	53	10
12	450 × 450 × 105	512 × 512× 53	0.88 × 0.88 × 3.0	53	10
13	450 × 450 × 105	512 × 512× 53	0.88 × 0.88 × 3.0	53	10
14	450 × 450 × 105	512 × 512× 53	0.88 × 0.88 × 3.0	53	10

The abdominal MRIs were normalized to [0,1] and resampled to a consistent volume size of 256 × 256 × 32, preserving the original in-plane resolution and maintaining the 3–5 mm slice thickness. During resampling, slices beyond the liver region were removed to retain the region of interest. Since the data were magnitude-only and lacked phase information, we simulated complex-valued MR images following a phase augmentation approach similar to that of Terpstra *et al* (2020). Specifically, synthetic phase maps were generated using sinusoidal functions and applied to the first respiratory bin (0%) of each subject’s 4D-MRI. These phase maps were then propagated to the remaining bins by performing inter-bin image registration with Elastix (Klein *et al* 2009), where the resulting DVFs were used to warp both the magnitude images and the phase maps. This process yielded a simulated set of complex-valued 4D MR images with respiratory-resolved phase modulation. 20 phase map sequences were generated for each subject. These phase maps were constructed using 3D sinusoidal functions with four spatial frequencies per Cartesian axis, randomly selected from 1.25 × 10^−3^ mm^−1^–2.50 × 10^−3^mm^−1^ (wavelengths of 800–400 mm), along with random phase shifts. To further augment the data, Gaussian noise was added separately to the real and imaginary parts of the complex-valued images at each respiratory phase. A total of 40 noise maps were generated using zero-mean noise with standard deviations randomly sampled from 3.0 × 10^−3^–5.0 × 10^−3^. From the augmented complex-valued volumes, undersampled *k*-space data were generated from them via NUFFT with radial readout trajectories (figure 2).

#### 2.4.2. Cardiac dataset

Cardiac *k*-space data from the publicly available OCMR dataset (Chen *et al* 2020) were used to validate the effectiveness of the proposed cMeta-INR framework. The MR scans were acquired on three Siemens MAGNETOM scanners—Prisma (3 T), Avanto (1.5 T), and Sola (1.5 T) (Siemens Healthineers, Munich, Germany)—using a gradient-echo steady-state free precession sequence, with variations in acquisition settings such as magnetic field strength, spatial resolution, and field of view. The data were mixed T2/T1-weighted, with TR ranging from 28.5 ms to 39.3 ms, TE from 1.41 ms to 1.53 ms, and flip angles from 33° to 70°. To serve as gold-standard references for evaluating the registration accuracy, 11 fully-sampled subjects were employed in our experiments. For each subject, 3D cine images were acquired in planar mode, with short-axis slices spanning from the base to the apex of the heart. A summary of the acquisition parameters is presented in table 2. Each cardiac cycle was temporally divided into multiple bins, with the initial bin (0%) corresponding to end-diastole state across all subjects.

**Table 2.** MR imaging parameters of the subjects in the cardiac dataset.

Subject	Field-of-view (mm^3^)	Volume Size	Resolution (mm^3^)	No. of slices	No. of Cardiac bins
1	760 × 285 × 80	384 × 144 × 10	1.98 × 1.98 × 8.0	10	25
2	720 × 309 × 112	384 × 156 × 14	1.88 × 1.98 × 8.0	14	22
3	720 × 270 × 80	320 × 120 × 10	2.25 × 2.25 × 8.0	10	30
4	760 × 285 × 96	384 × 144 × 12	1.98 × 1.98 × 8.0	12	19
5	760 × 285 × 96	384 × 144 × 12	1.98 × 1.98 × 8.0	12	25
6	760 × 285 × 96	384 × 144 × 12	1.98 × 1.98 × 8.0	12	21
7	600 × 233 × 66	288 × 112 × 11	2.08 × 2.08 × 6.0	11	18
8	760 × 285 × 96	384 × 144 × 12	1.98 × 1.98 × 8.0	12	25
9	720 × 270 × 96	320 × 120 × 12	2.25 × 2.25 × 8.0	12	23
10	760 ×308 × 112	384 × 156 × 14	1.98 × 1.98 × 8.0	14	22
11	800 × 314 × 48	448 × 176 × 6	1.78 × 1.78 × 8.0	6	28

To simulate undersampled *k*-space acquisitions of target volumes, complex-valued MR images were first reconstructed from fully-sampled *k*-space data using a reconstruction algorithm incorporating adaptive estimation of coil sensitivity maps (Walsh *et al* 2000). Similar to the abdominal dataset, all volumes were resampled to a consistent spatial resolution of 256 × 256 × 32 using trilinear interpolation, and intensity normalization was applied to each complex-valued image. Then, undersampled *k*-space data were generated from the reconstructed images via NUFFT with radial readout trajectories.

### 2.5. Implementation details

Due to the limited number of subjects in the abdominal and cardiac datasets, we adopted the same cross-validation and augmentation techniques as those used in KS-RegNet to evaluate the performance of our framework, and more details can be found in Shao *et al* (2022). However, unlike KS-RegNet, which was trained with a fixed spoke number/ undersampling factors, our framework employed random sampling of spoke numbers between 5 and 50 for the target volume acquisition during training (with the undersampling factor being consistent between slices for each volume). We used the NUFFT operator from the TorchKbNufft package (Muckley *et al* 2020) to obtain the undersampled *k*-space data. Each radial spoke contains 256 uniformly distributed sampling points. The radial trajectory was generated by incrementally rotating each subsequent spoke by the golden angle (111.25°) from a randomly selected initial azimuthal angle, which was resampled on-the-fly for each target volume during training.

We used VoxelMorph (Balakrishnan *et al* 2019) as the backbone for KS-RegNet, and a 5-layer MLP with sinusoidal activation functions as the INR (Sitzmann *et al* 2020), with the number of nodes set to (3, 256, 256, 256, 3). For the last year of the INR, no activation function was applied. The token-aware modulator consisted of three components: the Encoder used a Swin Transformer encoder block (Liu *et al* 2021) to produce 192-dimensional token features; the Attention part included a multi-head attention mechanism (4 heads) and pooling layers; and the Generator was a two-layer MLP that generated 256-dimensional modulation coefficients (*γ, β*). We used the Adam optimizer with a learning rate of 1 × 10^−5^ to train both KS-RegNet and cMeta-INR. The batch size was set to 4 for KS-RegNet and 1 for cMeta-INR. KS-RegNet was trained for 10 000 epochs, while cMeta-INR was trained with hyper-parameters ${N_{{\text{outer}}}} = 500$, ${N_{{\text{inner}}}} = 10$, and ${N_{{\text{adapt}}}} = 50$ (see algorithm 1).

For both datasets, we randomly selected a volume from each 4D-MRI as the source and another as the target for training KS-RegNet and cMeta-INR. During test-time adaptation, the first bin (0%) was used as the source and the others as target subjects. For each test subject, we simulated three levels of undersampling using 13, 9, and 5 spokes, corresponding to acceleration factors of approximately 31, 45, and 80, respectively. Unless specified, 13 spokes were used as the default setting.

### 2.6. Evaluation metrics

To evaluate the registration accuracy of cMeta-INR, we adopted two image-quality metrics: structural similarity index (SSIM) and relative error (RE), and three contour-based metrics: the 95th Hausdorff distance (HD95), Dice similarity coefficient (Dice), and center-of-mass error (COME).

The structural similarity between the deformed volume (estimated MRI) ${{{\hat I}}_{{\text{def}}}}$ and the reference (ground truth) volume ${I_{{\text{GT}}}}$ was quantified using the SSIM, defined as follows:

\begin{equation*}\text{SSIM} =\frac{\left( 2\hat{\mu}_{\text{def}} \mu_{\text{GT}} + c1 \right)\left( 2\,\text{cov}\left( \hat{I}_{\text{def}}, I_{\text{GT}} \right) + c2 \right)}{\left( \hat{\mu}_{\text{def}}^{2} + \mu_{\text{GT}}^{2} + c1 \right)\left( \hat{\sigma}_{\text{def}}^{2} + \sigma_{\text{GT}}^{2} + c2 \right)}\end{equation*}

where $\mu $ denotes the means, $\sigma $ denote the variances, ${\text{cov}}\left( \cdot \right)$ is the covariance, and ${c_1},{ }{c_2}$ are constants to avoid numerical instability.

The RE was quantified by measuring the intensity difference between the deformed volume ${{{\hat I}}_{{\text{def}}}}$ and the reference volume ${I_{{\text{GT}}}}$:

\begin{equation*}{\text{RE}} = \sqrt {\frac{{\sum\nolimits_N {{{\left( {{{\hat I}_{{\text{def}}}} - {I_{{\text{GT}}}}} \right)}^2}} }}{{\sum\nolimits_N {I_{{\text{GT}}}^2} }}} \times 100\% \end{equation*}

where $N$ is the number of volume voxels.

Let ${{{\hat M}}_{{\text{DS}}}} \in \left\{ {0,1} \right\}$ and ${M_{\text{T}}} \in \left\{ {0,1} \right\}$ represent the deformed source and ‘gold-standard’ target contour masks, respectively. The HD95 is calculated as:

\begin{equation*}{\text{HD}}95 = {\text{max}}\left\{ {{P_{95}}d\left( {{{\hat m}_{{\text{DS}}}},{M_{\text{T}}}} \right),{P_{95}}d\left( {{m_{\text{T}}},{{\hat M}_{{\text{DS}}}}} \right)} \right\},\end{equation*}

where ${P_{95}}$ is the 95-percentile operator, and $d\left( {{{\hat m}_{{\text{DS}}}},{M_{\text{T}}}} \right) = \mathop {\min }\limits_{{{\hat m}_{{\text{DS}}}} \in {{\hat M}_{{\text{DS}}}}} \|{\hat m_{{\text{DS}}}} - {M_{\text{T}}}\|$ is the distance from a deformed contour voxel ${{{\hat m}}_{{\text{DS}}}}$ to the ‘ground-truth’ mask ${\text{ }}{M_{\text{T}}}$. Similarly, $d\left( {{m_{\text{T}}},{{\hat M}_{{\text{DS}}}}} \right)$ is the distance from a ‘ground-truth’ contour voxel ${m_{\text{T}}}$ to the deformed source mask ${{{\hat M}}_{{\text{DS}}}}$.

The Dice measures the similarity between ${{{\hat M}}_{{\text{DS}}}}$ and ${M_{\text{T}}}$ by evaluating their spatial overlap, as follows:

\begin{equation*}{\text{Dice}} = \frac{{2\left| {{{{{\hat M}}}_{{\text{DS}}}} \cap {{\text{M}}_{{T}}}} \right|}}{{\left| {{{{{\hat M}}}_{{\text{DS}}}}} \right| + \left| {{{\text{M}}_{{T}}}} \right|}},\end{equation*}

where Dice = 0 means that ${{{\hat M}}_{{\text{DS}}}}$ and ${M_{\text{T}}}$ have no overlap, and Dice = 1 indicates that ${{{\hat M}}_{{\text{DS}}}}$ and ${M_{\text{T}}}$ are identical.

The COME measures the center-of-mass distance between the deformed source and ‘gold-standard’ contours, defined as:

\begin{equation*}{\text{COME}} =\left\| {{{\hat C}}_{{\text{DS}}}} - {{{C}}_{{T}}}\right\|_2,\end{equation*}

where ${{{\hat C}}_{{\text{DS}}}}$ and ${{\text{C}}_{{T}}}$ denote the centers-of-mass of ${{{\hat M}}_{{\text{DS}}}}$ and ${M_{\text{T}}}$, respectively.

To compute the evaluation metrics, anatomical contours were manually annotated in the MR datasets. In the abdominal dataset, liver tumors were segmented in the first respiratory bin (0%) by an experienced radiation oncologist and subsequently propagated to the remaining bins using DVFs generated by Elastix, which were also employed in the synthesis of complex-valued 4D-MRI data (section 2.4.1). For the cardiac dataset, the left ventricle was delineated across all cardiac bins for each subject, owing to its well-defined boundary in the short-axis view, providing a consistent and reliable structure for registration accuracy assessment.

### 2.7. Ablation study

To evaluate the effectiveness of the main components in the proposed cMeta-INR framework, we conducted four ablation experiments on both the abdominal and cardiac MRI datasets, via the following variants of cMeta-INR.

**Meta-INR** (baseline): it applies meta-learning to learn initialization parameters across training subjects, enabling fast test-time adaptation. The modulation parameters were generated by a CNN encoder without attention mechanisms and cohort-informed priors. The overall framework is similar to REINDIR (van Harten *et al* 2024), with necessary adaptations to fit our volumetric MRI estimation problem using limited *k*-space data (figure 2).

**Meta-INR_a** (structured modulation Meta-INR): Based on Meta-INR, the CNN encoder was replaced by the token-aware modulator (figure 1).

**Meta-INR_b** (cohort-informed Meta-INR_a): Deformation discrepancy loss (equation (2)) from a pre-trained KS-RegNet was introduced into Meta-INR_a, to guide the meta-learning process with cohort-informed priors.

**cMeta-INR** (full model): The embedding similarity loss (equation (4)) was further incorporated into Meta-INR_b to enforce semantic consistency between the deformed source and target embeddings in the latent space.

### 2.8. Comparison with other methods

We compared the performance of cMeta-INR with six related methods including iterative-based, population-based, INR-based, meta-learning-based, and hybrid approaches. Specifically, (1) **KS-Iterative**: a traditional iterative optimization-based method that estimates deformation in the *k*-space domain through gradient-based updates. (2) **KS-RegNet (**Shao *et al* 2022): an unsupervised, population-based registration model trained across subjects by minimizing loss in the *k*-space domain; (3) **KS-RegNet_T:** During test time, the KS-RegNet was further fine-tuned on each test case for 2000 epochs via unsupervised learning; (4)  **KS-INR:** training the INR from scratch for each case without any prior knowledge, by minimizing loss in the *k*-space domain; (5)  **KS-RegNet-INR (**Qian *et al* 2025): it first fits the INR to DVFs predicted by a pre-trained KS-RegNet, then refines it with a *k*-space loss; and (6) **Meta-INR:** as specified in section 2.7. These methods offered a comprehensive comparison with existing approaches across different learning paradigms.

## 3. Results

This section presented the experimental results to validate the effectiveness of the proposed cMeta-INR. We first conducted ablation and hyperparameter analyses to investigate the impact of key components and the three hyperparameters defined in equation (6) (section 3.1). Then, our method was compared with several related and representative approaches on two datasets (section 3.2). Finally, we analyzed the time complexity and robustness of the proposed framework (section 3.3).

### 3.1. Ablation study and hyperparameter analysis

#### 3.1.1. Ablation study

Table 3 presents the ablation results on the abdominal and cardiac datasets. Starting from the Meta-INR baseline, we observed consistent performance improvements as the proposed components were gradually integrated into the framework. On the abdominal dataset, when the CNN encoder was replaced with the token-aware modulator (Meta-INR_a), both SSIM and Dice showed improvement, while RE, HD95, and COME exhibited consistent reductions. Incorporating cohort-informed priors via DVFs estimated by KS-RegNet (Meta-INR_b) further enhanced performance, achieving a Dice of 0.775 ± 0.061. Finally, the full cMeta-INR model incorporating the embedding similarity loss achieved the best overall performance, with SSIM of 0.972 ± 0.012, RE of 9.35% ± 3.78%, Dice of 0.778 ± 0.056, HD95 of 3.00 ± 1.37 mm, and the lowest COME of 3.04 ± 1.48 mm.

**Table 3.** Comparison of mean (±s.d.) SSIM, RE, Dice, HD95, and COME metrics for the ablation study on two datasets. We adopted a multi-fold cross-validation strategy: for each fold, we used 11 abdominal cases or 9 cardiac cases for training, and the remaining 3 abdominal cases or 2 cardiac cases for testing. The spoke number used for *k*-space sampling is 13. The arrows point to the direction of improved accuracy. The best values are highlighted in bold.

Datasets	Methods			Mean (±s.d.)			
		SSIM↑	RE/%↓		Dice↑	HD95 (mm)↓	COME (mm)↓
Abdominal	Meta-INR(baseline)	0.965 ± 0.018	10.26 ± 3.02		0.762 ± 0.094	3.42 ± 1.52	3.13 ± 1.37
	Meta-INR_a	0.966 ± 0.011	10.08 ± 3.69		0.764 ± 0.088	3.25 ± 1.54	3.12 ± 1.52
	Meta-INR_b	0.968 ± 0.015	9.72 ± 4.13		0.775 ± 0.061	3.19 ± 1.92	3.08 ± 1.47
	**cMeta-INR**	**0.972 ± 0.012**	**9.35 ± 3.78**		**0.778 ± 0.056**	**3.00 ± 1.37**	**3.04 ± 1.48**

Cardiac	Meta-INR(baseline)	**0.976 ± 0.008**	8.78 ± 3.28		0.862 ± 0.056	4.72 ± 1.53	1.74 ± 1.18
	Meta-INR_a	0.978 ± 0.005	8.58 ± 3.21		0.875 ± 0.135	4.56 ± 2.33	1.51 ± 1.46
	Meta-INR_b	0.980 ± 0.006	7.86 ± 2.98		0.883 ± 0.122	4.09 ± 2.04	1.37 ± 1.17
	**cMeta-INR**	**0.983** ± **0.007**	**7.44** ± **2.76**		**0.894 ± 0.076**	**3.53 ± 1.26**	**1.32 ± 1.02**

On the cardiac dataset, the Dice increased from 0.862 ± 0.056 with the Meta-INR baseline to 0.875 ± 0.135 with structured modulation (Meta-INR_a), and further to 0.883 ± 0.122 with the incorporation of cohort-informed priors (Meta-INR_b). The proposed cMeta-INR achieved the highest Dice of 0.894 ± 0.076 and SSIM of 0.983 ± 0.007, together with the lowest RE of 7.44% ± 2.76%, HD95 of 3.53 ± 1.26 mm, and COME of 1.32 ± 1.02 mm, demonstrating consistent improvements in anatomical fidelity and boundary precision. These results provide strong evidence for the effectiveness of incorporating structured token-aware modulation, population-level priors, and semantic consistency into the proposed framework.

#### 3.1.2. Hyperparameter analysis

**Figure 4.**
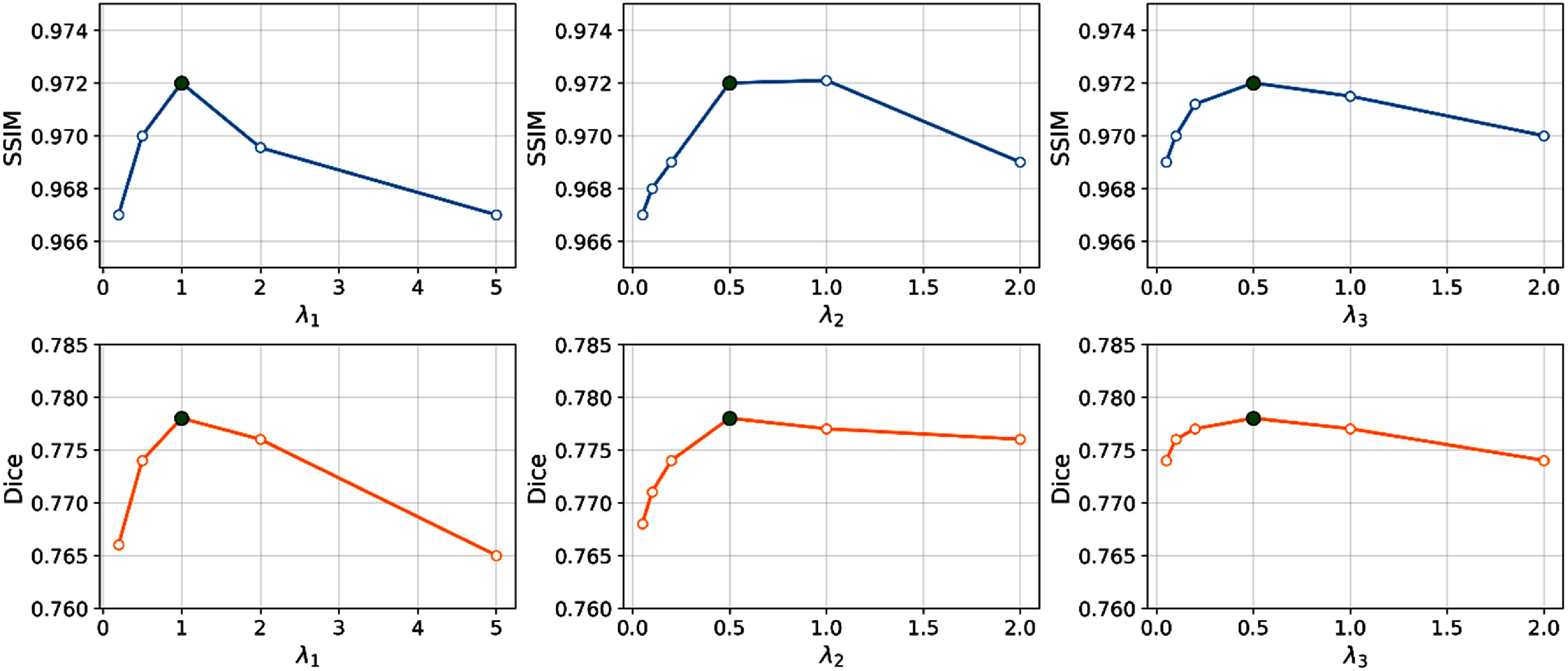
Hyperparameter analysis of the loss weighting coefficients. The top row shows SSIM variations with respect to ${\lambda _1}$, ${\lambda _2}$, and ${\lambda _3}$, and the bottom row shows the corresponding Dice results. In each experiment, two parameters were fixed at (${\lambda _1} = 1$.0, ${\lambda _2} = 0.5,{\text{ }}{\lambda _3} = 0.5$), and the remaining one was varied.

We conducted a detailed analysis of the hyperparameters (${\lambda _1},{ }{\lambda _2},{ }{\lambda _3}$) in the total loss function of step B. In each experiment, two parameters were fixed at their default values, while the third was varied within a predefined range to evaluate the performance of cMeta-INR on the abdominal dataset. Specifically, ${\lambda _1}$ was tested in [0.2, 0.5, 1.0, 2.0, 5.0], ${\lambda _2}$ in [0.05, 0.1, 0.2, 0.5, 1.0, 2.0], and ${\lambda _3}$ in [0.05, 0.1, 0.2, 0.5, 1.0, 2.0]. Model performance was quantitatively evaluated using SSIM and Dice as shown in figure 4.

Both SSIM and Dice exhibit a similar trend with respect to the three hyperparameters. As shown in figure 4, model performance first increases and then decreases as the weight of each term increases. For ${\lambda _1}$, both SSIM and Dice reach their peak when ${\lambda _1}$ = 1.0, after which the performance gradually declines. A similar trend can be seen for ${\lambda _2}$, where the best results are obtained around ${\lambda _2}$ =0.5. For ${\lambda _3}$, the performance remains relatively stable within the range of 0.2–0.5 and slightly drops when the value exceeds 1.0. The optimal values of the three hyperparameters were thus determined to be ${\lambda _1} = 1$.0, ${\lambda _2} = 0.5,{ }$ and ${\lambda _3} = 0.5$, which were used in all subsequent experiments.

### 3.2. Comparison with other methods

#### 3.2.1. Qualitative comparison of different methods on two datasets

**Figure 5.**
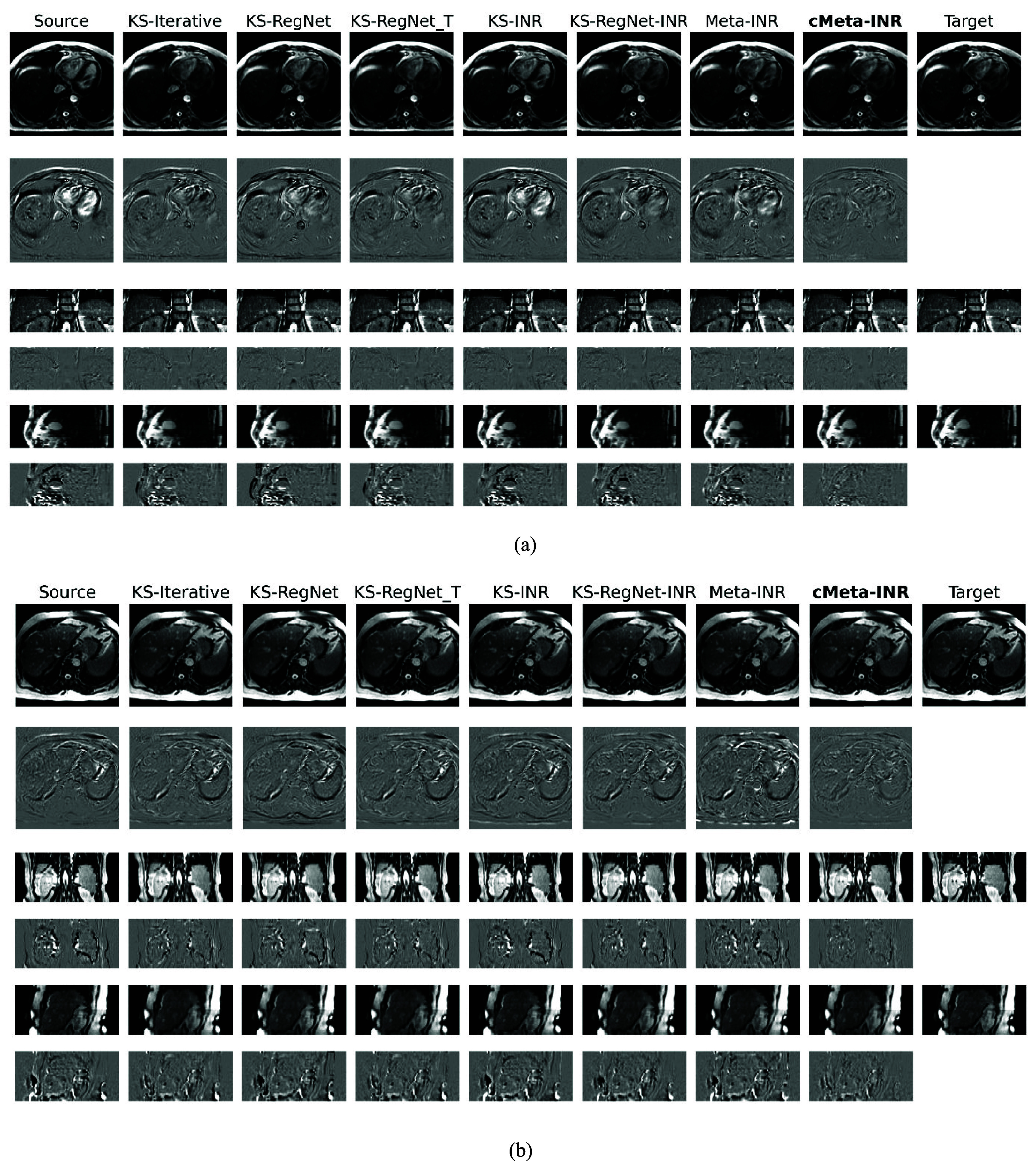
Comparison of abdominal MR images solved by different methods. Subfigures (a) and (b) present the images from different subjects in the axial, coronal, and sagittal views (odd rows), and the differences between these images and the target images (even rows). The spoke number used for *k*-space sampling is 13.

**Figure 6.**
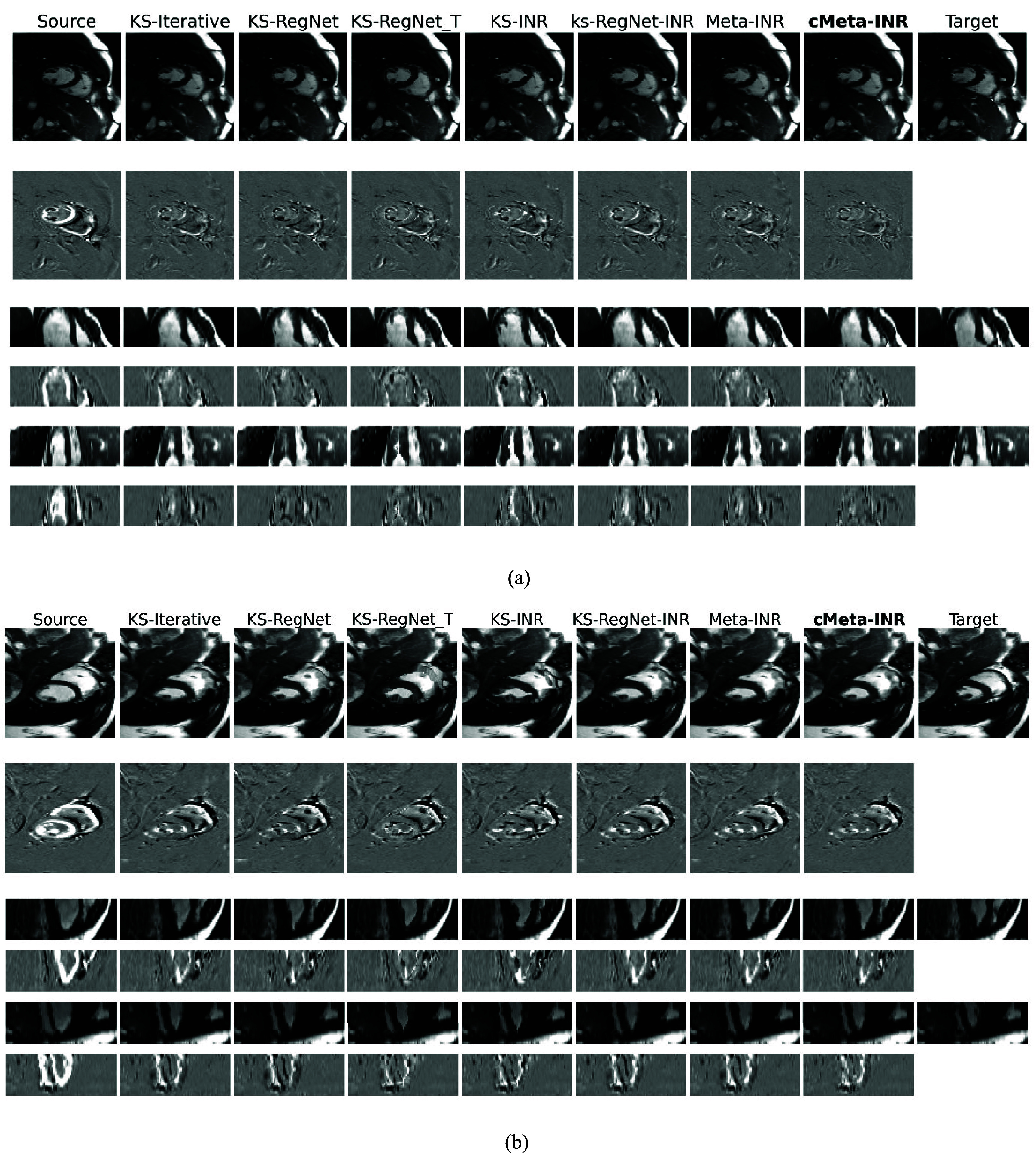
Comparison of cardiac MR images by different methods. Subfigures (a) and (b) present the images from different subjects in the axial, coronal, and sagittal views (odd rows), and the differences between these images and the target images (even rows). The spoke number used for *k*-space sampling is 13.

Figures 5 and 6 present qualitative registration results from the cardiac (two subjects) and abdominal (two subjects) datasets, respectively. Each figure included the source image, the target image, the deformed source images produced by various methods, and their difference maps for visual assessment of the registration accuracy. On the abdominal dataset, KS-iterative, a representative conventional method based on gradient-driven iterative optimization of DVFs, achieved competitive performance. KS-RegNet, trained across subjects and applied directly to new ones, exhibited residual errors near organ boundaries, indicating its limited ability to capture subject-specific variations. Further test-time fine-tuning by KS-RegNet_T produced improved alignment, but boundary artifacts persisted, particularly in the axial plane. Although KS-INR enabled case-specific optimization, its representation capacity remained inferior to KS-RegNet under limited iteration settings. KS-RegNet-INR enhanced organ-level alignment by leveraging prior DVFs, achieving smoother contours in the coronal view. Meta-INR improved structural consistency across views and further suppressed residuals near the liver dome. Among all methods, cMeta-INR demonstrated the most accurate alignment, with minimal residual differences.

On the cardiac dataset, the difference maps revealed that KS-iterative and KS-RegNet exhibited noticeable residuals around the apex. Although KS-RegNet_T introduced subject-specific fine-tuning and produced slight improvements over KS-RegNet, the limited single-subject data proved insufficient to fully optimize the network parameters. Training INR from scratch reduced overall misalignment but introduced more high-frequency artifacts in the difference maps and had residual deformation errors near the right ventricular wall. By leveraging prior-guided DVFs, KS-RegNet-INR yielded smoother results, though small mismatches still persisted at anatomical boundaries. Meta-INR, designed to accelerate INR’s test-time optimization, resulted in improved accuracy relative to the vanilla KS-INR, particularly in regions such as the septum and apex. Notably, cMeta-INR had the most clean difference maps across cardiac cases and reduced artifacts along anatomical boundaries. cMeta-INR outperformed the other methods, achieving more precise, anatomically consistent registrations with better structural boundaries and fewer artifacts.

#### 3.2.2. Quantitative comparison of different methods on two datasets

Table 4 summarizes the quantitative results of different registration methods on the abdominal and cardiac datasets, evaluated using the SSIM, RE, Dice, HD95, and COME metrics. NR refers to unregistered results, serving as a reference. Across both datasets, the proposed cMeta-INR consistently outperformed the comparative methods. On the abdominal dataset, cMeta-INR achieved the highest Dice of 0.778 ± 0.056, surpassing KS-RegNet and Meta-INR, indicating improved anatomical overlap. It also yielded the lowest COME (3.04 ± 1.48 mm), reflecting more precise boundary alignment and motion estimation. The improvements over population-based methods (KS-RegNet, KS-RegNet_T) and other KS-INR variants suggested that incorporating both population priors and structured modulation significantly enhanced accuracy. Table 4 also shows the results on the cardiac dataset, where cMeta-INR attained a Dice of 0.894 ± 0.076, outperforming all other methods. It also achieved the lowest COME of 1.32 ± 1.02 mm, confirming more accurate deformation estimation in the presence of complex cardiac motion. Wilcoxon signed-rank tests with Bonferroni corrections were conducted to compare cMeta-INR with each of the other five methods. All *p*-values were Bonferroni-corrected for multiple comparisons, where each *p*-value was multiplied by the number of compared methods, with the significance threshold remaining at 0.05 after correction. In most cases, the resulting *p*-values were below 10^−3^.

**Table 4.** Comparison of mean (±s.d.) SSIM, RE, Dice, HD95, and COME between different methods on two datasets. We adopted a multi-fold cross-validation strategy: for each fold, we used 11 abdominal cases or 9 cardiac cases for training, and the remaining 3 abdominal cases or 2 cardiac cases for testing. The spoke number used for *k*-space sampling is 13. The arrows point to the direction of improved accuracy. Wilcoxon signed-rank tests with Bonferroni corrections were performed between cMeta-INR and the other methods. The best values are shown in bold.

Datasets	Methods			Mean (±s.d.)					*p*-values		
		SSIM↑	RE/%↓	Dice↑	HD95↓	COME↓	SSIM	RE	Dice	HD95	COME
Abdominal	NR	0.935 ± 0.027	19.60 ± 5.73	0.674 ± 0.157	6.10 ± 2.04	4.60 ± 2.37	**—**	**—**	**—**	**—**	**—**
	KS-Iterative	0.951 ± 0.016	11.02 ± 3.45	0.749 ± 0.130	3.52 ± 1.98	3.44 ± 1.62	<10^−3^	<10^−3^	<10^−3^	<10^−3^	<10^−3^
	KS-RegNet	0.945 ± 0.018	12.54 ± 4.23	0.741 ± 0.116	3.67 ± 2.12	3.51 ± 1.73	<10^−3^	<10^−3^	<10^−3^	<10^−3^	<10^−3^
	KS-RegNet_T	0.950 ± 0.019	11.43 ± 3.41	0.747 ± 0.128	3.62 ± 2.05	3.35 ± 1.66	<10^−3^	<10^−3^	<10^−3^	<10^−3^	<10^−3^
	KS-INR	0.943 ± 0.018	11.58 ± 3.38	0.732 ± 0.082	3.83 ± 1.62	3.96 ± 2.04	<10^−3^	<10^−3^	<10^−3^	<10^−3^	<10^−3^
	KS-RegN-INR	0.958 ± 0.021	10.99 ± 3.16	0.759 ± 0.106	3.39 ± 1.93	3.21 ± 1.25	<10^−3^	<10^−3^	<10^−3^	<10^−3^	0.002
	Meta-INR	0.965 ± 0.018	10.26 ± 3.02	0.762 ± 0.094	3.42 ± 1.52	3.13 ± 1.37	<10^−3^	<10^−3^	0.001	<10^−3^	0.007
	**cMeta-INR**	**0.972 ± 0.012**	**9.35 ± 3.78**	**0.778 ± 0.056**	**3.00 ± 1.37**	**3.04 ± 1.48**	**—**	**—**	**—**	**—**	**—**

Cardiac	NR	0.956 ± 0.007	13.45 ± 4.46	0.771 ± 0.124	6.24 ± 2.58	2.47 ± 1.66			**—**	**—**	**—**
	KS-Iterative	0.979 ± 0.009	8.86 ± 4.13	0.869 ± 0.025	4.54 ± 1.82	1.57 ± 1.28	0.002	<10^−3^	<10^−3^	<10^−3^	<10^−3^
	KS-RegNet	0.976 ± 0.007	9.64 ± 4.21	0.871 ± 0.053	4.65 ± 1.48	1.71 ± 1.33	<10^−3^	<10^−3^	<10^−3^	<10^−3^	<10^−3^
	KS-RegNet_T	0.979 ± 0.009	9.00 ± 3.57	0.873 ± 0.072	4.53 ± 1.92	1.58 ± 1.25	<10^−3^	<10^−3^	<10^−3^	<10^−3^	<10^−3^
	KS-INR	0.972 ± 0.006	8.34 ± 4.37	0.855 ± 0.066	5.01 ± 2.05	1.83 ± 1.53	<10^−3^	<10^−3^	<10^−3^	<10^−3^	<10^−3^
	KS-RegN-INR	0.978 ± 0.007	8.26 ± 4.12	0.875 ± 0.064	4.49 ± 1.38	1.50 ± 1.06	<10^−3^	0.005	<10^−3^	<10^−3^	0.001
	Meta-INR	0.976 ± 0.008	8.78 ± 3.28	0.862 ± 0.056	4.72 ± 1.53	1.74 ± 1.18	<10^−3^	<10^−3^	<10^−3^	<10^−3^	<10^−3^
	**cMeta-INR**	**0.983 ± 0.007**	**7.44** ± **2.76**	**0.894 ± 0.076**	**3.53 ± 1.26**	**1.32 ± 1.02**	**—**	**—**	**—**	**—**	**—**

### 3.3. Time complexity analysis and robustness tests

#### 3.3.1. Time complexity analysis

**Figure 7.**
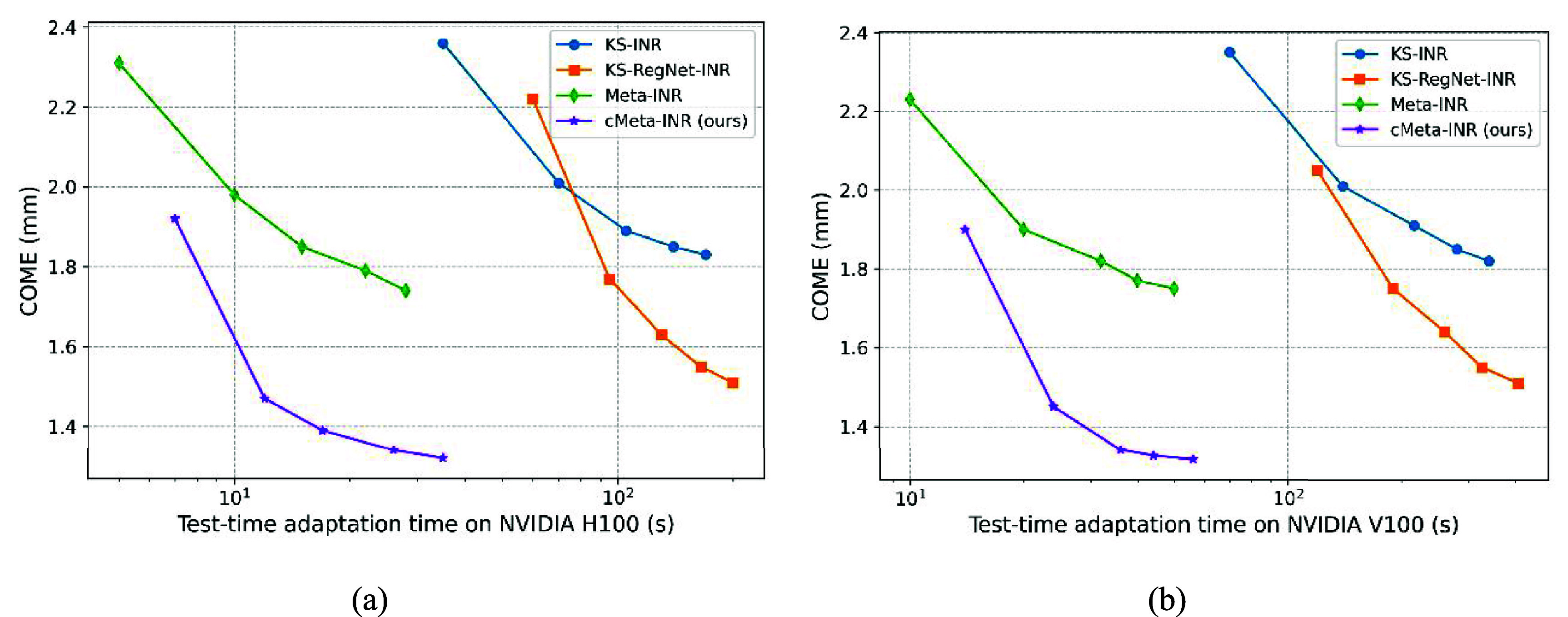
Test-time adaptation performance comparison among four methods: KS-INR, KS-RegNet-INR, Meta-INR, and the proposed cMeta-INR. The *x*-axis represents the total test-time adaptation time (*s*) under different iteration settings, and the *y*-axis shows the corresponding COME (mm). Each point represents the average result on the cardiac dataset. Experiments were conducted on (a) NVIDIA H100 GPU and (b) NVIDIA V100 GPU.

Test-time adaptation was constrained by the optimization time required for each case. To mitigate this issue, we introduced meta-learning and cohort-informed priors to reduce inference overhead. In this section, we compared the test-time optimization efficiency of four methods: KS-INR from scratch, KS-RegNet-INR, Meta-INR, and the proposed cMeta-INR. The models were trained under various iteration settings: [100, 200, 300, 400, 500] steps for KS-INR; [2000/100, 2000/200, 2000/300, 2000/400, 2000/500] for KS-RegNet-INR; [10, 20, 30, 40, 50] for Meta-INR; and [50/10, 50/20, 50/30, 50/40, 50/50] for cMeta-INR, where 2000/100 indicates that step A ran for 2000 epochs and step B for 100 epochs. Since step A was significantly faster than step B, we fixed the number of epochs for step A for both KS-RegNet-INR (2000) and cMeta-INR (50). We reported the average COME and runtime for one case on the cardiac dataset under different iteration settings. All experiments were performed on an NVIDIA H100 GPU (figure 7(a)) and an NVIDIA V100 GPU (figure 7(b)) to compare runtime across different hardware platforms.

As shown in figure 7, all methods exhibited improved performance (lower COME) with increasing test-time adaptation steps, but with different efficiency profiles. KS-INR from scratch required the longest adaptation time to reach acceptable performance, while KS-RegNet-INR converged more quickly by leveraging prior deformation guidance. Meta-INR achieved substantially lower runtime by initializing the network with meta-learned parameters, yet its accuracy remained suboptimal. The proposed cMeta-INR outperformed all other methods across different iteration settings, achieving lower COME with significantly reduced adaptation time. These results demonstrated that incorporating both meta-learning and cohort-informed priors enabled faster and more accurate test-time optimization. The runtime of different models on the NVIDIA V100 GPU was approximately twice that on the H100 GPU.

#### 3.3.2. Test under varying undersampling factors

By leveraging population-level information during training and case-specific optimization at test time, the cMeta-INR model is designed to generalize to unseen subjects with different undersampling levels. In addition to the default 13-spoke test setting, we evaluated the accuracy of our method under two more undersampling configurations with 9 and 5 radial spokes, corresponding to higher acceleration factors. As shown in tables 5 and 6, our method achieved stable performance across these settings, demonstrating its robustness to variations in sampling factors.

**Table 5.** Comparison of mean (±s.d.) SSIM, RE, Dice, HD95, and COME between different methods on the abdominal dataset for the 9- and 5-spoke sampling scenarios. We adopted a multi-fold cross-validation strategy: for each fold, we used 11 abdominal cases for training, and the remaining 3 cases for testing. The arrows point to the direction of improved accuracy. Wilcoxon signed-rank tests with Bonferroni corrections were performed between cMeta-INR and the other methods, all yielding *p* values < 10^−3^. The best values are shown in bold.

Number of spokes	Abdominal Dataset/methods	SSIM↑	RE/%↓	Dice↑	HD95 (mm)↓	COME (mm)↓
5	KS-Iterative	0.945 ± 0.013	11.54 ± 3.05	0.741 ± 0.123	3.97 ± 1.54	3.92 ± 1.35
	KS-RegNet	0.932 ± 0.016	12.77 ± 3.33	0.732 ± 0.125	4.12 ± 1.77	4.15 ± 1.77
	KS-RegNet_T	0.941 ± 0.017	12.23 ± 2.16	0.737 ± 0.108	4.00 ± 1.98	4.06 ± 1.64
	KS-INR	0.940 ± 0.014	12.01 ± 3.15	0.723 ± 0.113	4.26 ± 2.16	4.22 ± 2.31
	KS-RegNet-INR	0.950 ± 0.016	11.51 ± 2.83	0.748 ± 0.082	3.96 ± 2.08	3.83 ± 1.36
	Meta-INR	**0.962 ± 0.013**	10.47 ± 3.02	0.751 ± 0.153	3.82 ± 1.63	3.70 ± 1.54
	**cMeta-INR**	0.961 ± 0.012	**9.82 ± 2.14**	**0.763 ± 0.101**	**3.43 ± 1.06**	**3.39 ± 1.37**

9	KS-Iterative	0.948 ± 0.014	11.38 ± 3.15	0.747 ± 0.105	3.88 ± 2.53	3.51 ± 1.63
	KS-RegNet	0.940 ± 0.017	12.63 ± 3.26	0.736 ± 0.108	3.96 ± 2.32	3.96 ± 1.76
	KS-RegNet_T	0.946 ± 0.015	12.00 ± 2.92	0.744 ± 0.103	3.78 ± 2.17	3.68 ± 1.84
	KS-INR	0.941 ± 0.018	11.87 ± 3.93	0.727 ± 0.108	4.24 ± 2.23	4.16 ± 2.21
	KS-RegNet-INR	0.952 ± 0.016	11.35 ± 2.16	0.751 ± 0.104	3.71 ± 2.16	3.57 ± 1.62
	Meta-INR	0.963 ± 0.017	10.31 ± 2.16	0.758 ± 0.107	3.68 ± 1.34	3.44 ± 2.11
	**cMeta-INR**	**0.968 ± 0.014**	**9.48 ± 2.76**	**0.772 ± 0.101**	**3.27 ± 0.99**	**3.25 ± 1.23**

**Table 6.** Comparison of mean (±s.d.) SSIM, RE, Dice, HD95, and COME between different methods on the cardiac dataset for the 9- and 5-spoke sampling scenarios. We adopted a multi-fold cross-validation strategy: for each fold, we used 9 cardiac cases for training, and the remaining 2 cases for testing. The arrows point to the direction of improved accuracy. Wilcoxon signed-rank tests with Bonferroni corrections were performed between cMeta-INR and the other methods, all yielding *p*-values < 10^−3^. The best values are shown in bold.

Number of Spokes	Cardiac Dataset/ Methods	SSIM↑	RE/%↓	Dice↑	HD95 (mm)↓	COME (mm)↓
5	KS-Iterative	0.967 ± 0.005	9.01 ± 3.52	0.858 ± 0.084	4.72 ± 1.63	1.90 ± 1.54
	KS-RegNet	0.963 ± 0.005	9.95 ± 4.86	0.849 ± 0.092	4.86 ± 1.78	2.07 ± 1.82
	KS-RegNet_T	0.968 ± 0.007	9.81 ± 3.73	0.855 ± 0.078	4.65 ± 2.48	1.93 ± 1.77
	KS-INR	0.962 ± 0.007	9.00 ± 2.31	0.843 ± 0.122	5.13 ± 2.22	1.92 ± 1.58
	KS-RegNet-INR	0.973 ± 0.005	8.56 ± 3.20	0.859 ± 0.094	4.63 ± 2.13	1.63 ± 0.52
	Meta-INR	0.971 ± 0.006	9.14 ± 325	0.849 ± 0.952	4.90 ± 2.06	1.78 ± 1.68
	**cMeta-INR**	**0.979** ± **0.006**	**8.22** ± **4.12**	**0.872 ± 0.081**	**4.08 ± 1.87**	**1.43 ± 1.23**

9	KS-Iterative	0.972 ± 0.007	8.92 ± 4.35	0.866 ± 0.090	4.59 ± 1.63	1.76 ± 1.45
	KS-RegNet	0.965 ± 0.006	9.71 ± 4.28	0.860 ± 0.085	4.77 ± 2.05	1.98 ± 1.26
	KS-RegNet_T	0.971 ± 0.006	9.63 ± 3.64	0.868 ± 0.065	4.58 ± 1.74	1.62 ± 1.27
	KS-INR	0.969 ± 0.008	8.48 ± 3.50	0.854 ± 0.124	5.07 ± 2.80	1.86 ± 1.63
	KS-RegNet-INR	0.975 ± 0.006	8.33 ± 3.22	0.873 ± 0.093	4.55 ± 1.58	1.52 ± 1.30
	Meta-INR	0.977 ± 0.007	8.94 ± 4.01	0.856 ± 0.085	4.74 ± 1.62	1.75 ± 1.12
	**cMeta-INR**	**0.980** ± **0.006**	**7.87 ± 3.64**	**0.884 ± 0.068**	**3.85 ± 1.38**	**1.40 ± 1.01**

Tables 5 and 6 present the evaluation results of different methods for varying undersampling conditions (i.e., number of spokes = 5 and 9). For both abdominal and cardiac datasets, cMeta-INR achieved the highest SSIM, Dice, and the lowest RE, HD95, and COME values under both undersampling configurations, showing low variance and stable accuracy and demonstrating its robustness to severe information loss from highly undersampled *k*-space data. In addition, all Wilcoxon signed-rank tests of the three metrics comparing cMeta-INR with each of the other five methods yielded p-values below 10^−3^.

## 4. Discussion

Medical image registration plays a crucial role in many clinical applications, particularly in motion-resolved dynamic imaging, where accurate alignment of temporally resolved volumes is essential for motion analysis and diagnosis. In this study, we developed a registration-driven method to map prior, fully-sampled MRI volumes to new MRI volumes based on undersampled *k*-space data, leveraging the high-quality prior information for real-time imaging. Population-based registration models offered fast inference but often failed to account for case-specific anatomical or acquisitional variability. In contrast, test-time optimized models based on INRs provided better accuracy, but required longer computation time due to their per-case optimization nature. To address these limitations, we proposed cMeta-INR, a cohort-informed meta-learning framework that integrated population-level priors and structured token-aware modulation. The proposed approach combined the efficiency of population-based meta-learning methods with the flexibility/accuracy of case-specific optimization, achieving precise registration with significantly fewer adaptation steps. By leveraging shared anatomical knowledge during meta-training, cMeta-INR achieved fast, accurate, and robust per-case adaptation at test time. cMeta-INR introduced three key innovations to enhance both the registration accuracy and the optimization efficiency of INRs. First, the token-aware modulator enabled fine-grained spatial adaptation by injecting localized anatomical information through token attention into the structured modulation process. Unlike conventional global modulation schemes (REINDIR, for instance), this design captured spatial heterogeneity more effectively, thereby improving the model’s adaptability to case-specific anatomical variations. Second, the incorporation of cohort-informed guidance via DVFs predicted by a pre-trained KS-RegNet provided a strong population-level prior learned through a deeper network. This not only stabilized meta-learning but also accelerated convergence by offering informed initialization, mitigating the need to learn from scratch. Third, the introduction of an embedding similarity loss (equation (4)) promoted consistency between the deformed source and target representations in the latent space. This latent alignment further refined spatial correspondence and enhanced the semantic coherence of the predicted deformations. Finally, these components worked synergistically to improve the convergence speed and registration accuracy of the model. The benefit of each design was quantitatively validated through the ablation study, demonstrating their contributions to the overall framework.

Although relatively small training cohorts may introduce potential overfitting and generalization constraints in the population-based meta-learning initialization, these limitations are inherently alleviated by the patient-specific test-time optimization, which further refines the model for each new case. This adaptive optimization reduces the dependence on large training datasets and helps maintain stable performance. In the qualitative evaluation (figures 5 and 6), cMeta-INR produced sharper structural boundaries and cleaner difference maps than the comparison methods, indicating more accurate anatomical alignment and fewer residual mismatches. These improvements were particularly evident in anatomically complex regions, such as the septum and apex in cardiac datasets, and the liver boundary and left upper abdomen (e.g. spleen-adjacent areas) in abdominal datasets, where non-rigid motion is prominent. In the quantitative analysis (table 4), cMeta-INR achieved higher Dice and lower HD95 and COME values compared to other methods on both cardiac and abdominal datasets. While cMeta-INR required per-case test-time adaptation, its meta-learned initialization significantly reduced optimization time (∼35 s) relative to conventional INRs (figure 7(a)). Furthermore, the model maintained stable performance under higher undersampling ratios (e.g., 9 and 5 spokes), confirming its robustness to *k*-space sparsity and acquisition degradation (tables 5 and 6). Collectively, these results suggest that cMeta-INR generalizes well and remains effective under diverse acquisition protocols, supporting its potential for clinical deployment in real-world scenarios.

Although cMeta-INR demonstrated superior performance and efficiency in registration-driven real-time volumetric MRI estimation through limited *k*-space data, several potential limitations remain and warrant further investigation in future work. First, cMeta-INR relies on a pre-trained KS-RegNet to provide population-level deformation priors, which may limit its adaptability in scenarios where the test data significantly deviate from the training distribution. In this study, the performance gain of the proposed method (cMeta-INR) over the test-time-only methods primarily derives from the pretrained INR template, which provides a better initialization for subsequent test-time optimization. On the other hand, its advantage over the population-based methods is mainly due to the additional case-specific test-time optimization, which helps to further fine-tune the registration results. Due to the improvements from both directions, the proposed cMeta-INR method is expected to outperform the existing baselines, as we did not observe any testing case where cMeta-INR underperformed the baseline methods. However, we did observe in limited scenarios that the relative advantage of cMeta-INR over baseline methods is small. For these cases, the image intensity distribution of the testing case usually has a large difference from the training cases. Due to such domain gaps, the benefit of meta-learning-based initialization is reduced, and the registration accuracy is mainly driven by the test-time optimization itself. In such scenarios, the performance of cMeta-INR generally remains comparable to baseline methods that rely on test-time optimization. Second, the synthesis of complex-valued 4D-MRI using simulated phase maps follows strategies that have been validated with real data (Zhu *et al* 2018, Terpstra *et al* 2020). Both phase and magnitude are propagated through the same Elastix-derived DVFs, and the generated data are self-consistent and minimally affected by registration inaccuracies. Even so, this remains an approximate strategy, and further validation with real clinical complex-valued data is warranted. Third, in our current clinical setting, undersampled *k*-space data of moving anatomy are difficult to obtain. Following existing *k*-space data generation strategies to train/test deep learning models (Terpstra *et al* 2020, Shao *et al* 2022, Xiao *et al* 2023), we employed NUFFT-based simulation to generate *k-*space data from fully sampled 4D MRIs, with the fully sampled images also readily serving as ‘ground truth’ for evaluation. All compared methods were evaluated using the same data generation and sampling scheme to ensure fairness and consistency. Previous studies of deep learning models trained on such simulated data found they generalized well to real clinical data (Zhu *et al* 2018, Terpstra *et al* 2020, Hossbach *et al* 2023). The INR framework can readily adapt to other *k*-space sampling strategies, including those used in parallel imaging, provided that the *k*-space data for each instance are acquired within a short time frame to be considered as ‘motion-free’. Future studies are warranted to evaluate cMeta-INR using real *k*-space data acquired with diverse sampling schemes. Fourth, although our approach achieves fast test-time adaptation, the overall pipeline involves multiple components—including meta-learning, structured modulation, and external priors—that introduce additional training complexity. Future research could explore simplification through model distillation, structure pruning, or adaptive inference mechanisms to further reduce computational overhead (Tran *et al* 2022, Valverde *et al* 2024, Zhang *et al* 2024). Although case-specific test-time optimization has been reduced to tens of seconds by cMeta-INR, the runtime still exceeds the time constraint of real-time imaging for simultaneous delivery adaptation (∼0.5 s). Further acceleration could be achieved by (i) DVF dimensionality reduction via motion modeling (e.g. PCA-based low-rank subspaces) (Fransson *et al* 2021) and (ii) backpropagation-free, on-the-fly test-time inference (Niu *et al* 2024), etc. In addition, this study focuses on evaluating the imaging performance of the proposed method in terms of reconstruction/registration accuracy and efficiency, demonstrating that cMeta-INR is an effective approach to enable real-time volumetric MR imaging. The registration accuracy indicates the potential of cMeta-INR in achieving accurate real-time motion tracking of treatment targets. It would be valuable to further investigate and extend this technique in future studies to broader clinical applications and to evaluate its performance on downstream tasks in adaptive radiotherapy, such as dose calculation and accumulation.

## 5. Conclusions

In this work, we presented cMeta-INR, a cohort-informed meta-learning framework for registration-driven real-time volumetric MRI estimation using limited *k*-space data. By integrating structured modulation and population-guided priors, our method achieved accurate and efficient test-time adaptation. Extensive experiments on both abdominal and cardiac 4D-MRI datasets demonstrated the superiority of cMeta-INR over population-based, INR-based, and meta-learning approaches, in terms of registration accuracy, inference efficiency, and robustness to undersampling variations. These results highlight the potential of combining meta-learning with cohort priors for real-time, high-fidelity medical image registration and estimation.

## Data Availability

The OCMR cardiac dataset can be accessed at www.ocmr.info/download/. The data cannot be made publicly available upon publication due to legal restrictions preventing unrestricted public distribution. The data that support the findings of this study are available upon reasonable request from the authors.
